# Propagation of Blood Function Errors to the Estimates of Kinetic Parameters with Dynamic PET

**DOI:** 10.1155/2011/234679

**Published:** 2010-11-28

**Authors:** Yafang Cheng, İmam Şamil Yetik

**Affiliations:** Department of Electrical Engineering, Medical Imaging Research Center, Illinois Institute of Technology, Chicago, IL 60616-3793, USA

## Abstract

Dynamic PET, in contrast to static PET, can identify temporal variations in the radiotracer concentration. Mathematical modeling of the tissue of interest in dynamic PET can be simplified using compartment models as a linear system where the time activity curve of a specific tissue is the convolution of the tracer concentration in the plasma and the impulse response of the tissue containing kinetic parameters. Since the arterial sampling of blood to acquire the value of tracer concentration is invasive, blind methods to estimate both blood input function and kinetic parameters have recently drawn attention. Several methods have been developed, but the effect of accuracy of the estimated blood function on the estimation of the kinetic parameters is not studied. In this paper, we present a method to compute the error in the kinetic parameter estimates caused by the error in the blood input function. Computer simulations show that analytical expressions we derive are sufficiently close to results obtained from numerical methods. Our findings are important to observe the effect of the blood function on kinetic parameter estimation, but also useful to evaluate various blind methods and observe the dependence of kinetic parameter estimates to certain parts of the blood function.

## 1. Introduction

Positron emission tomography (PET) is a functional imaging modality to observe the physiological processes in the body. To conduct a PET scan, positron-emitting radioisotopes, as a tracer, are injected into the living subject (usually into blood circulation). When a positron encounters and annihilates an electron, it emits two gamma rays in reverse directions which will be sensed at two detectors at roughly the same time. Hence it is possible to locate the source along the line of response using a scanner around the subject. The data from the detector is then used to reconstruct an image of the subject [1].

Temporal variation of the tracer concentration can be obtained through dynamic imaging so that the physiological function of the subject can be tracked more accurately. Such a kinetic approach is commonly used in other imaging modalities (e.g., dynamic contrast enhanced MRI [2]) and photodynamic therapy [3]. Dynamic PET, in contrast to static PET, can provide kinetic parameters that are related to physiologic information and is a useful tool for various clinical and research applications [4–8]. A three-compartment model is commonly used in fluoro-2-deoxy-D-glucose (FDG) studies that we use for tumor analysis to simplify the kinetic model of the tracer molecule of interest. In this model, the input *C*_*p*_(*t*) is the tracer concentration in the plasma, and the output is the time activity curve (TAC). The TACs are obtained by averaging the activity of a known region of interest. Let *f*(*t*) denote the TAC of a specific tissue, then the relation between *f*(*t*) and the impulse response of a region *h*(*t*) can be obtained using diffusion equations.

**Figure 1:**
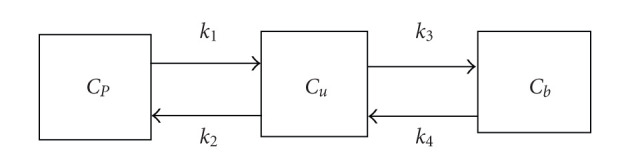
Three compartments used to model the transfer of the tracer between physical compartments and chemical states.

The differential equations that describe the FDG three-compartment model (Figure 1) are expressed as follows:

(1) $$\begin{array}{cc} \frac{\text{d}C_{u}\left(t\right)}{\text{d}t} & =k_{1}C_{p}\left(t\right)-\left(k_{2}+k_{3}\right)C_{u}\left(t\right)+k_{4}C_{b}\left(t\right),\\ \frac{\text{d}C_{b}\left(t\right)}{\text{d}t} & =k_{3}C_{u}\left(t\right)-k_{4}C_{b}\left(t\right), \end{array}$$

where *C*_*p*_(*t*) denotes the tracer concentration in the plasma assumed to be spatially constant, *C*_*u*_(*t*) denotes the concentration of unbounded tracer, *C*_*b*_(*t*) is the concentration of the bounded tracer, and *t* denotes the time coordinate. The solution is [8]

(2) $$\begin{array}{cc} C_{u}\left(t\right) & =\frac{k_{1}}{c-d}\left[\left(k_{4}-d\right)e^{-dt}+\left(c-k_{4}\right)e^{-ct}\right]*C_{p}\left(t\right),\\ C_{b}\left(t\right) & =\frac{k_{1}k_{3}}{c-d}\left[e^{-dt}-e^{-ct}\right]*C_{p}\left(t\right), \end{array}$$

for *t* > 0, where “∗” denotes convolution and

(3) $$\begin{array}{c} d=\frac{\left(k_{2}+k_{3}+k_{4}\right)}{2}-\frac{\sqrt{{\left(k_{2}+k_{3}+k_{4}\right)}^{2}-4k_{2}k_{4}}}{2},\\ c=\frac{\left(k_{2}+k_{3}+k_{4}\right)}{2}+\frac{\sqrt{{\left(k_{2}+k_{3}+k_{4}\right)}^{2}-4k_{2}k_{4}}}{2}. \end{array}$$

We use the following transformations as commonly done [9] since the transformed parameters are more suitable for our mathematical model:

(4) $$\begin{array}{cc} a & =\frac{k_{1}}{2\Delta }\left(k_{2}-k_{3}-k_{4}+\Delta \right),\\ b & =\frac{k_{1}}{2\Delta }\left(-k_{2}+k_{3}+k_{4}+\Delta \right),\\ c & =0.5\left(k_{2}+k_{3}+k_{4}+\Delta \right),\\ d & =0.5\left(k_{2}+k_{3}+k_{4}-\Delta \right),\\ \Delta & =\left|\sqrt{{\left(k_{2}+k_{3}+k_{4}\right)}^{2}-4k_{2}k_{4}}\right| \end{array}$$

with inverse transforms

(5) $$\begin{array}{cc} k_{1} & =a+b,\\ k_{2} & =\frac{ac+bd}{a+b},\\ k_{3} & =\frac{ab{\left(c-d\right)}^{2}}{\left(a+b\right)\left(ac+bd\right)},\\ k_{4} & =\frac{cd\left(a+b\right)}{ac+bd}. \end{array}$$

Then, for the three-compartment tissue modeling, the relation between *f*(*t*) and the impulse response *h*(*t*) containing the kinetic parameters is

(6) $$\begin{array}{c} f\left(t\right)=h\left(t\right)*C_{p}\left(t\right), \end{array}$$

where *h*(*t*) is

(7) $$\begin{array}{c} h\left(t\right)=ae^{-ct}+be^{-dt}. \end{array}$$

Estimation of the kinetic parameters *k*_1_, *k*_2_, *k*_3_, and *k*_4_ for three-compartment tissue modeling based on *f*(*t*) requires that the blood function *C*_*p*_(*t*) is known. The classical method of arterial sampling to obtain the blood function has several disadvantages: it requires well-trained medical personnel and poses a vital risk to the subject. Therefore, blind methods are developed to estimate the kinetic parameters of the tissue response without knowing input function. In such methods, the input function is estimated along with the kinetic parameters of the tissue impulse model of interest, and thus scale parameters such as *k*_1_, *k*_2_, *k*_3_, and *k*_4_ and any expression using these parameters can only be estimated in a relative sense instead of an absolute one. Several studies have been done in the field, such as maximum likelihood, cross-relation methods, and several others, see [10–13] and references therein.

Since we have discrete measurements at times *t* = Δ*t* · *n* for noisy TAC measurements we can use the following model

(8) $$\begin{array}{c} f^{\left(i\right)}\left(n\right)=h^{\left(i\right)}\left(n\right)*C_{p}\left(n\right)+\varepsilon^{\left(i\right)}\left(n\right), \end{array}$$

which can be written as

(9) $$\begin{array}{c} {\vec{f}}^{\left(i\right)}=H^{\left(i\right)}\vec{C_{p}}+{\vec{\varepsilon }}^{\left(i\right)}, \end{array}$$

where *H*^(*i*)^ is the convolution matrix of the impulse response of the tissue for region *i*, *C*_*p*_ denotes the vector of the blood function, and ${\vec{\varepsilon }}^{\left(i\right)}$ is the noise vector. Stacking different regions of interest together, we can write the equations in the following form

(10) $$\begin{array}{c} \vec{F}=H\vec{C_{p}}+\vec{\varepsilon }. \end{array}$$

We can estimate the kinetic parameters and the blood function by minimizing the following cost function:

(11) $$\begin{array}{c} R={||\vec{F}-\hat{H}\vec{{\hat{C}}_{p}}||}^{2}. \end{array}$$

Several methods for blind kinetic parameter estimation has been proposed, but no study has shown the effect of the errors in the estimated blood on the estimation of kinetic parameters except for our preliminary work [14]. In this paper, we develop a method that can compute this effect in an efficient manner. Our results can be used to calculate the error in the kinetic parameter estimates stemming from the errors in the blood function that is used. Our derivations is based on the implicit function theorem previously used for static PET [15] and the Runge-Kutte methods [16].

Although, these errors in the parameters can be found performing a separate optimization for each of the error combinations in the blood that we want to study, this is a very time-consuming method, considering that the optimization procedure is iterative. This is especially important when we are interested in pixel by pixel kinetic parameter estimation, and/or when the space of the erroneous blood functions we want to analyze is large. Based on the results of this paper, the optimization needs to be performed only once, and the error propagation can be calculated very fast based on this single optimization.

Our major contribution in this paper is to construct a mathematical model to derive the error in the kinetic parameter estimates caused by the error in estimation of the blood input function. Our results are conceptually important to observe the effect of the error in the blood function on kinetic parameter estimation, and also practically useful to evaluate various blind methods and observe the dependence of kinetic parameter estimates to certain parts of the blood function such as the peak and tail part. In Section 2, we illustrate the derivation of the mathematical model.  Section 3 includes computer simulations that validate the analytical results. Conclusions are presented in Section 4.

## 2. Derivation of the Error Propagation for Three-Compartment Tissue Modeling

In this section, we explain how we can calculate the errors in the kinetic parameters for three-compartment tissue modeling due to the error in the blood function. We assume that a unique solution for the blood estimates, at least locally, exists. The estimates of the kinetic parameters $\hat{a}$, $\hat{b}$, $\hat{c}$, $\hat{d}$ are

(12) $$\begin{array}{c} \left[\hat{a},\hat{b},\hat{c},\hat{d}\right]=\arg\;\underset{\hat{a},\hat{b},\hat{c},\hat{d}}{\min\;\;}R\left(\left[\hat{a},\hat{b},\hat{c},\hat{d}\right],{\hat{C}}_{p}\right). \end{array}$$

Our goal is to obtain the errors in the kinetic parameters Δ*a*, Δ*b*, Δ*c*, and Δ*d* stemming from the error in the blood function Δ*C*_*p*_. The first step in this calculation is to calculate the derivatives of the implicit estimator with respect to the elements of *C*_*p*_(*t*) at a fixed *C*_*p*_(*t*) point. The second step is to accumulate the error sequentially to derive the ultimate error based on the first step.

The first step can be performed by using the chain rule and implicit function theorem [15].

For a solution *a*, *b*, *c*, *d* that minimizes the cost function the partial derivatives of cost function with respect to the kinetic parameters is zero

(13) $${0=\frac{\partial }{\partial a}R\left(\left[\hat{a},\hat{b},\hat{c},\hat{d}\right],{\hat{C}}_{p}\right)|}_{a=\hat{a}}$$

and three similar equations. Let us define an implicit function which will be convenient for the application of chain rule. We need to use the derivatives of the following equation to derive the expression of the derivatives of the implicit estimator with respect to the elements of *C*_*p*_(*t*) at a fixed *C*_*p*_(*t*) point

(14) $$\begin{array}{c} \left[\hat{a},\hat{b},\hat{c},\hat{d}\right]=g\left({\hat{C}}_{p}\right)={\left[g_{1}\left({\hat{C}}_{p}\right),g_{2}\left({\hat{C}}_{p}\right),g_{3}\left({\hat{C}}_{p}\right),g_{4}\left({\hat{C}}_{p}\right)\right]}^{\prime } \end{array}$$

that maps the ${\hat{C}}_{p}$ into an estimate $\left[\hat{a},\hat{b},\hat{c},\hat{d}\right]$. By applying the chain rule to this equation, we can differentiate the above two equations with respect to ${\hat{C}}_{p}$ and obtain

(15) $$\begin{array}{cc} 0 & =\frac{\partial^{2}}{\partial a^{2}}R\left(g\left({\hat{C}}_{p}\right),{\hat{C}}_{p}\right)\frac{\partial }{\partial {\hat{C}}_{p}}g_{1}\left({\hat{C}}_{p}\right)\\ & \;+\frac{\partial^{2}}{\partial a\partial b}R\left(g\left({\hat{C}}_{p}\right),{\hat{C}}_{p}\right)\frac{\partial }{\partial {\hat{C}}_{p}}g_{2}\left({\hat{C}}_{p}\right)\\ & \;+\frac{\partial^{2}}{\partial a\partial c}R\left(g\left({\hat{C}}_{p}\right),{\hat{C}}_{p}\right)\frac{\partial }{\partial {\hat{C}}_{p}}g_{3}\left({\hat{C}}_{p}\right)\\ & \;+\frac{\partial^{2}}{\partial a\partial d}R\left(g\left({\hat{C}}_{p}\right),{\hat{C}}_{p}\right)\frac{\partial }{\partial {\hat{C}}_{p}}g_{4}\left({\hat{C}}_{p}\right)\\ & \;+\frac{\partial^{2}}{\partial a\partial {\hat{C}}_{p}}R\left(g\left({\hat{C}}_{p}\right),{\hat{C}}_{p}\right), \end{array}$$

and similar equations for *b*, *c*, and *d* can be obtained easily.

Thus we derive the derivative expression that we are interested in,

(16) $$\begin{array}{cc} \left[\begin{array}{c} \frac{\partial g_{1}\left({\hat{C}}_{p}\right)}{\partial {\hat{C}}_{p_{n}}}\\ \frac{\partial g_{2}\left({\hat{C}}_{p}\right)}{\partial {\hat{C}}_{p_{n}}}\\ \frac{\partial g_{3}\left({\hat{C}}_{p}\right)}{\partial {\hat{C}}_{p_{n}}}\\ \frac{\partial g_{4}\left({\hat{C}}_{p}\right)}{\partial {\hat{C}}_{p_{n}}} \end{array}\right] & ={\left[\begin{array}{cccc} \frac{\partial^{2}}{\partial a^{2}}R & \frac{\partial^{2}}{\partial a\partial b}R & \frac{\partial^{2}}{\partial a\partial c}R & \frac{\partial^{2}}{\partial a\partial d}R\\ \frac{\partial^{2}}{\partial b\partial a}R & \frac{\partial^{2}}{\partial b^{2}}R & \frac{\partial^{2}}{\partial b\partial c}R & \frac{\partial^{2}}{\partial b\partial d}R\\ \frac{\partial^{2}}{\partial c\partial a}R & \frac{\partial^{2}}{\partial c\partial b}R & \frac{\partial^{2}}{\partial c^{2}}R & \frac{\partial^{2}}{\partial c\partial d}R\\ \frac{\partial^{2}}{\partial d\partial a}R & \frac{\partial^{2}}{\partial d\partial b}R & \frac{\partial^{2}}{\partial d\partial c}R & \frac{\partial^{2}}{\partial d^{2}}R \end{array}\right]}^{-1}\\ & \;\times \left[\begin{array}{c} -\frac{\partial^{2}}{\partial a\partial {\hat{C}}_{p_{n}}}R\\ -\frac{\partial^{2}}{\partial b\partial {\hat{C}}_{p_{n}}}R\\ -\frac{\partial^{2}}{\partial c\partial {\hat{C}}_{p_{n}}}R\\ -\frac{\partial^{2}}{\partial d\partial {\hat{C}}_{p_{n}}}R \end{array}\right]. \end{array}$$

Because of the presence of ∂, *b*  ,*c*, and *d* in the terms $\partial \text{a}/\partial {\hat{C}}_{p_{n}}$, $\partial b/\partial {\hat{C}}_{p_{n}}$, $\partial c/\partial {\hat{C}}_{p_{n}}$, and $\partial d/\partial {\hat{C}}_{p_{n}}$, we cannot simply integrate $(\partial \text{a}/\partial {\hat{C}}_{p_{n}})\Delta {\hat{C}}_{p_{n}}$, $(\partial b/\partial {\hat{C}}_{p_{n}})\Delta {\hat{C}}_{p_{n}}$, $(\partial c/\partial {\hat{C}}_{p_{n}})\Delta {\hat{C}}_{p_{n}}$, and $(\partial d/\partial {\hat{C}}_{p_{n}})\Delta {\hat{C}}_{p_{n}}$ to obtain the errors in the kinetic parameter estimates. However, we can calculate $\partial \text{a}/\partial {\hat{C}}_{p_{n}}$, $\partial b/\partial {\hat{C}}_{p_{n}}$, $\partial c/\partial {\hat{C}}_{p_{n}}$, and $\partial d/\partial {\hat{C}}_{p_{n}}$ at a fixed point of ${\hat{C}}_{p_{n}}$, and a sequential procedure can be applied to calculate a new value of *a*, *b*, *c*, and *d*, and we can use them to evaluate $\partial \text{a}/\partial {\hat{C}}_{p_{n}}$, $\partial b/\partial {\hat{C}}_{p_{n}}$, $\partial c/\partial {\hat{C}}_{p_{n}}$, and $\partial d/\partial {\hat{C}}_{p_{n}}$ at the next *C*_*p*_ value until the complete range of Δ*C*_*p*_ is covered. This procedure can be performed by methods such as Runge-Kutte methods, predictor-corrector method and Richardson extrapolation.

(17) $$\begin{array}{cc} s_{1,i} & =hu_{i}\left(C_{p_{n}},a,b,c,d\right),\\ s_{2,i} & =hu_{i}\left(C_{p_{n}}+\frac{h}{2},a+\frac{s_{1,1}}{2},b+\frac{s_{1,2}}{2},c+\frac{s_{1,3}}{2},d+\frac{s_{1,4}}{2}\right),\\ s_{3,i} & =hu_{i}\left(C_{p_{n}}+\frac{h}{2},a+\frac{s_{2,1}}{2},b+\frac{s_{2,2}}{2},c+\frac{s_{2,3}}{2},d+\frac{s_{2,4}}{2}\right),\\ s_{4,i} & =hu_{i}\left(C_{p_{n}}+h,a+s_{3,1},b+s_{3,2},c+s_{3,3},d+s_{3,4}\right),\\ y_{i}\left(C_{p_{n}}+h\right) & =y_{i}\left(C_{p_{n}}\right)+\frac{s_{1,i}}{6}+\frac{s_{2,i}}{3}+\frac{s_{3,i}}{3}+\frac{s_{4,\text{i}}}{6}, \end{array}$$

where *k*_*i*_ = *y*_*i*_(*C*_*p*_*n*__), *i* = 1,2, 3,4 for *a*, *b*, *c*, and *d*, and *h* is the small step size we define according to the demand on accuracy and speed.

This completes the calculation of the errors in *a*, *b*, *c*, and *d* stemming from the error in estimation of *C*_*p*_*n*__. To summarize the expressions derived in this section provide the errors in the kinetic parameters Δ*a*, Δ*b*, Δ*c*, Δ*d* stemming from errors in the blood Δ*C*_*p*_. The number of steps to derive the ultimate error depends on the step size you specify in different cases to meet the requirement of the accuracy. Then we can use (3) to transform *a*, *b*, *c*, and *d* to obtain *k*_1_, *k*_2_, *k*_3_, and *k*_4_. These equations show the computational advantage of the proposed method of calculating the error compared to optimization approach. In the optimization approach, we need to calculate the cost function and the gradients for each of the iterations, whereas here we need to calculate (17) for only a few steps.

The detailed computation of our mathematical model can be seen in the appendix.

## 3. Computer Simulations

We apply the proposed mathematical model to simulated dynamic PET data with *t* = [0.25, 0.5, 0.75, 1.0, 1.25, 1.5, 1.75, 2.0, 2.5, 3.0, 3.5, 7.0, 10.0, 15.0, 20.0, 30.0, 60.0, 90.0, 120.0] min, where the ending time of each frame is used, and three sets of kinetic parameters for liver, background, and tumor: the first set of liver: *k*_1_ = 0.8, *k*_2_ = 0.98, *k*_3_ = 0.012, *k*_4_ = 0.017 (*a* = 0.79, *b* = 0.01, *c* = 0.9922, *d* = 0.0168); the second set of background: *k*_1_ = 0.01, *k*_2_ = 0.05, *k*_3_ = 0.001, *k*_4_ = 0.0001 (*a* = 0.0098, *b* = 0.00019683, *c* = 0.0510, *d* = 0.000098035); the third set of tumor: *k*_1_ = 0.598, *k*_2_ = 0.680, *k*_3_ = 0.05, *k*_4_ = 0.001 (*a* = 0.5569, *b* = 0.0411, *c* = 0.7301, *d* = 0.00093142). These values are selected to produce a realistic case in [9]. The blood function is produced using the model [12]:

(18) $$\begin{array}{c} C_{p}\left(t\right)=\left(b_{1}t-b_{2}-b_{3}\right)e^{\lambda_{1}t}+b_{2}e^{\lambda_{2}t}+b_{3}e^{\lambda_{3}t} \end{array}$$

with typical values from [12]

(19) $$\left[b_{1},b_{2},b_{3},\lambda_{1},\lambda_{2},\lambda_{3}\right]=\left[900,22,21,-4.1,-0.12,-0.01\right]{\min\;}^{-1}.$$

**Figure 2:**
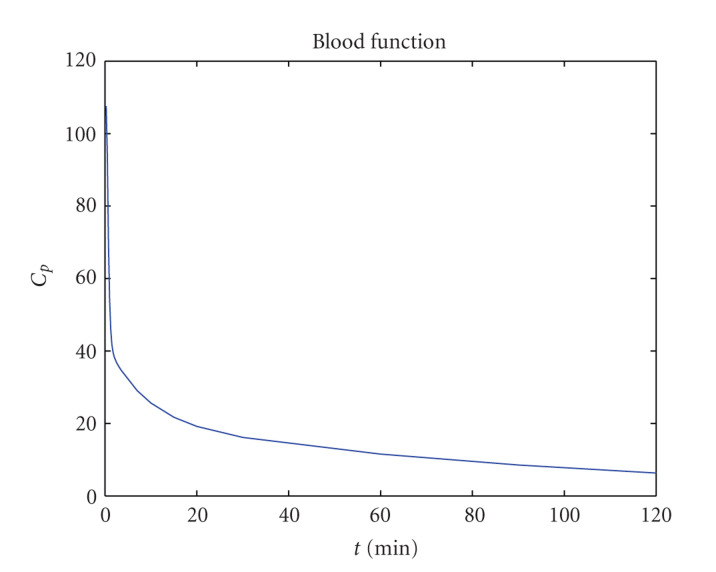
The input blood function.

The input blood function can be seen in Figure 2.

**Figure 3:**
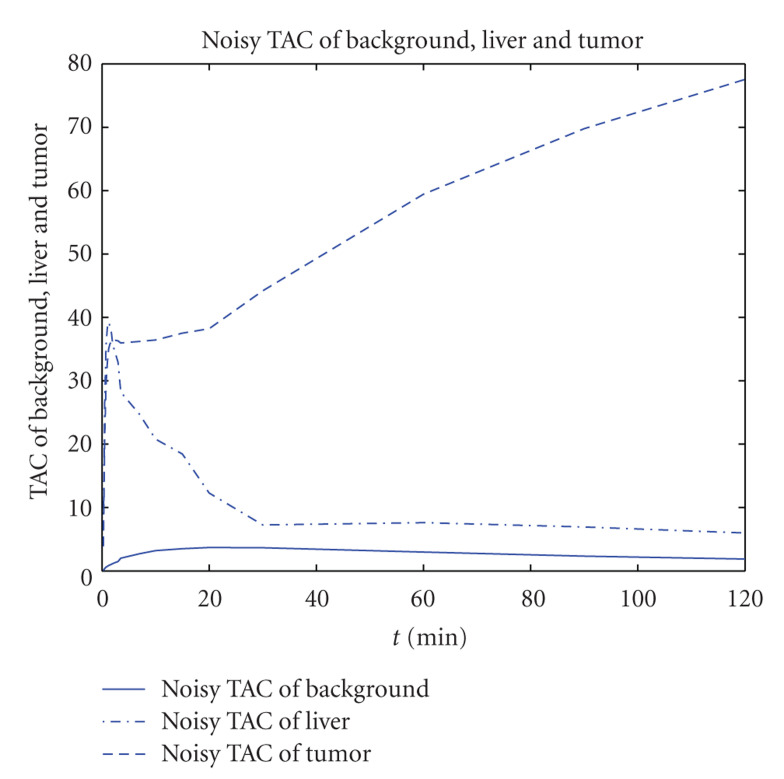
The observed noisy TAC for background, liver, and tumor.

We simulate a noisy TAC value as follows:

(20) $$\begin{array}{c} F_{\text{noisy}}=F_{\text{original}}\cdot \left(1+N\right), \end{array}$$

where *N* is Gaussian Noise with variance 0.01. The noisy TAC model for liver, background, and tumor can be seen in Figure 3. This model adds noise with the power proportional to the activity level to mimic a realistic case.

We have performed two sets of experiments. For these three experiments, we set an error margin for *C*_*p*_*n*__ and compare the propagated errors in *k*_1_, *k*_2_, *k*_3_, and *k*_4_ of liver and tumor using the derived expressions and optimization. Optimization is a numerical method using the conjugate gradient method (CGM).

**Figure 4:**
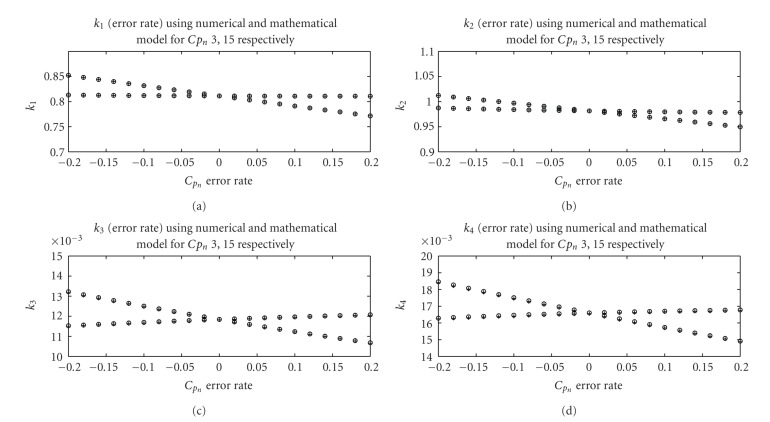
Comparison between the estimated *k*_1_, *k*_2_, *k*_3_, and *k*_4_ of liver using the derived expressions and numerical method for a range of erroneous blood functions. A single sample out of 19 samples of *C*_*p*_*n*__ has an error. The results are given for two random samples.

First, we test the mathematical model in one dimension and assume that one of the 19 samples of *C*_*p*_*n*__ has an error −20% to 20% defined as (*C*_*p*_*n*__(error) − *C*_*p*_*n*__(true))/*C*_*p*_*n*__(true).  Figure 4 shows a comparison of the results from the derived expressions and optimization for two arbitrary samples of *C*_*p*_. We observe that the derived expressions provide very accurate approximations of the the errors in *k*_1_, *k*_2_, *k*_3_, and *k*_4_ of liver.

**Figure 5:**
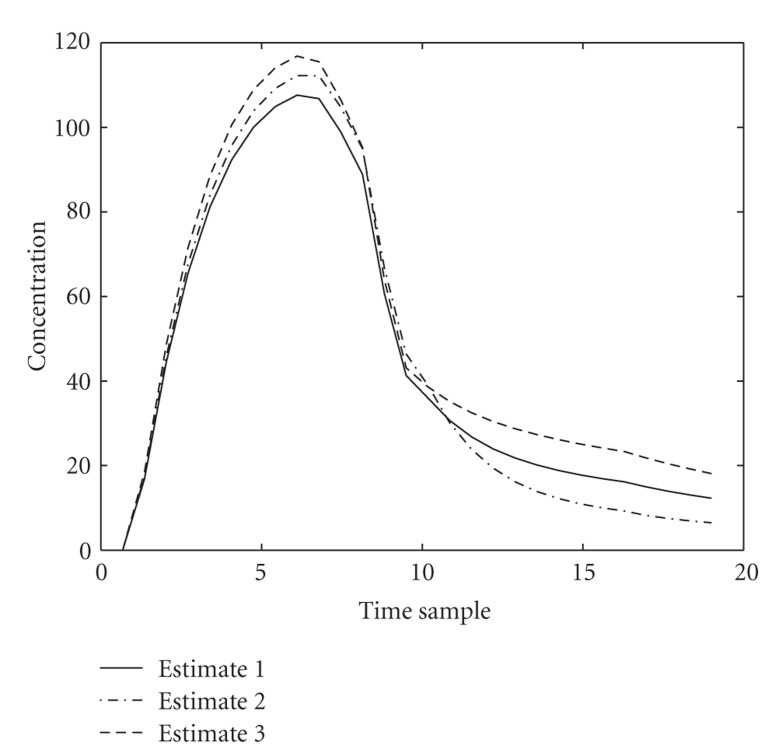
Three compartments used to model the transfer of the tracer between physical compartments and chemical states.

For several of the blind methods, the error in the blood function is not usually confined in a single sample. To illustrate this fact, we have performed a simple simulation where we have estimated the blood function with three different noise realizations.  Figure 5 shows that, the initial peak, transition part, and tail section of the estimated blood function is affected differently motivating this type of simulation. Therefore, in our second experiment, we use a blood function with multiple erroneous samples. The blood function is divided into two parts: (i) the initial peak and (ii) the tail part. Based on this grouping three cases of errors are considered: Case I: all samples; Case II: initial peak with samples 1 to 13; Case III: tail part with samples 14 to 19 are erroneous. All erroneous samples have the same error rate ranging from −20% to 20% with the three cases defined before.

**Figure 6:**
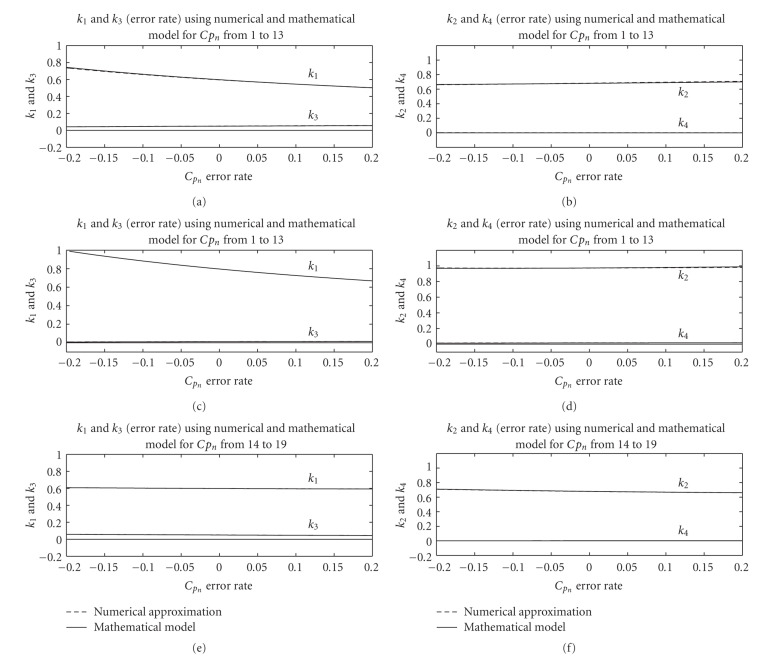
Comparison between the estimated *k*_1_, *k*_2_, *k*_3_, and *k*_4_ of liver using the derived expressions and numerical approximation for a range of erroneous blood functions. Top two figures show results when all samples of the blood are erroneous, (a,b)/(c,d) when the initial peak is erroneous, and (e,f) when the tail part is erroneous.

**Figure 7:**
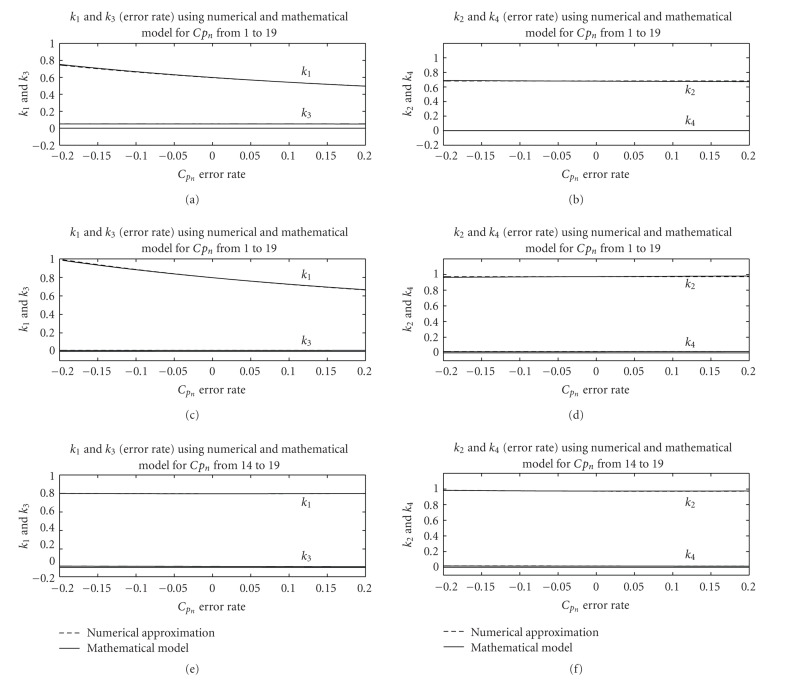
Comparison between the estimated *k*_1_, *k*_2_, *k*_3_, and *k*_4_ of tumor using the derived expressions and numerical approximation for a range of erroneous blood functions. Top two figures show results when all samples of the blood are erroneous, middle two when the initial peak is erroneous, and the bottom two when the tail part is erroneous.

Figures 6 and 7 show that the results from the derived expressions are very close to ones obtained from numerical optimization.

The following items summarize the effect of the blood function error to the final kinetic parameter estimates.

- For Case I, when all 19 samples have the same error rate from −20% to 20%, we can see that the changes in *k*_2_, *k*_3_, and *k*_4_ are negligibly small while change in *k*_1_ is relatively large. *k*_1_ of liver drops from 1.0093 to 0.6728, *k*_1_ of background drops from 0.0126 to 0.0084, *k*_1_ of tumor drops from 0.7599 to 0.5067. And *k*_2_, *k*_3_, and *k*_4_ data in the Figures 5, 6, and 7 are flat lines. This can be explained with (7) and (8) where scaling in the blood function would not affect *c* and *d* but inversely scale *a* and *b*.
- For Case II, we observe that *k*_1_ and *k*_3_ deviate from the true value considerably, *k*_4_ also deviates from the true value but not as much, while *k*_2_ almost remains the same, indicating that the error in the initial peak affects the estimation of kinetic parameter *k*_1_, *k*_3_, and *k*_4_ more than *k*_2_. *k*_1_ of liver decreases from 1.0122 to 0.6766, *k*_2_ of liver increases from 0.9784 to 0.9866, *k*_3_ of liver increases from 0.0110 to 0.0129, *k*_4_ of liver decreases from 0.0195 to 0.0155. *k*_1_ of background decreases from 0.0132 to 0.0081, *k*_2_ of background decreases from 0.0594 to 0.0433, *k*_3_ of background increases from 0.0001 to 0.0017, *k*_4_ of background increases from −0.0215 to 0.0055. *k*_1_ of tumor decreases from 0.7455 to 0.5142, *k*_2_ of tumor increases from 0.6545 to 0.7124, *k*_3_ of tumor increases from 0.0449 to 0.0552, and *k*_4_ of tumor decreases from 0.0024 to 0.0003.
- For Case III, we observe a different effect; the error in the tail part affects the estimation of kinetic parameter *k*_3_ most, *k*_4_ to a lesser degree but does not affect *k*_1_ and *k*_2_. *k*_1_ of liver decreases from 0.8141 to 0.8118, *k*_2_ of liver decreases from 0.9941 to 0.9815, *k*_3_ of liver decreases from 0.0156 to 0.0095, *k*_4_ of liver decreases from 0.0186 to 0.0155. *k*_1_ of background increases from 0.0097 to 0.0105, *k*_2_ of background increases from 0.0397 to 0.0601, *k*_3_ of background increases from 0.0008922 to 0.0009358, *k*_4_ of background decreases from 0.0025 to −0.0013. *k*_1_ of tumor decreases from 0.6176 to 0.6029, *k*_2_ of tumor decreases from 0.7173 to 0.6690, *k*_3_ of tumor decreases from 0.0588 to 0.0446, and *k*_4_ of tumor increases from 0.0005 to 0.0018.

In addition to the figures, Table 1 lists the relations between the error rate of *C*_*p*_ and the percentage of changes in estimation of the parameters *k*_1_, *k*_2_, *k*_3_, and *k*_4_ of the liver in three cases defined before.

Our conclusion of the relation between the blood function error and the error on the kinetic parameters can be summarized as follows:

- We can see that the error in the initial peak of the blood input function affects the estimation of kinetic parameter *k*_1_, *k*_3_, and *k*_4_ more than *k*_2_. The parameter *k*_3_ changes 12.5% when error rate of *C*_*p*_ becomes 10% while *k*_1_ changes 10% and *k*_4_ changes 4%.
- And the error in the tail part affects the estimation of kinetic parameter *k*_3_ most, *k*_4_ second but does not affect *k*_1_ and *k*_2_. The parameter *k*_3_ will change 10% when error rate of *C*_*p*_ becomes 10% while *k*_1_ will change 5%.
- If the overall blood input function has almost the same error rate, we find that the error in the parameters *k*_2_, *k*_3_, and *k*_4_ is negligibly small while *k*_1_ changes relatively large. In this case, only the parameter *k*_1_ will change 10% when error rate of *C*_*p*_ becomes 10%.

Table 2 lists the relations between the error rate of *C*_*p*_ and the percentage of changes in estimation of the parameters *k*_1_, *k*_2_, *k*_3_, and *k*_4_ of the tumor in three cases defined before. Similar conclusion can be drawn from the data of the table.

These simulation results show that the derived expressions provide a very accurate approximation of the errors in the kinetic parameters, and several useful observations related to the effect of the blood function on the kinetic parameter estimates can be made.

## 4. Conclusion

Blind identification is recently studied to estimate the kinetic parameters for the compartment without a known blood input function since the arterial sampling of blood input function involves vital risks, requires trained personnel, is not comfortable for the patient in clinical applications, and is difficult to perform in small animals. There are several solutions for blind identification: maximum likelihood methods, cross-relation method, mixture analysis method, and factor analysis of dynamic structures.

Despite several blind methods, how an erroneous blood function affects the final parameter values has not been studied. In this paper, we have derived mathematical expressions that quantify how errors in the blood estimate propagate into errors in the kinetic parameter estimates. Our model is constructed on the base of the implicit function theorem and the Runge-Kutte methods. We first derive the derivative of the kinetic parameters with respect to blood input function at a fixed point. Then we implement the Runge-Kutte approximation to calculate the accumulated errors affected by the gradually increased error in blood input function. The accuracy of the mathematical model can be modified by adjusting the number of steps in the Runge-Kutte approximation as desired.

Computer simulations show that the proposed mathematical model can yield accurate estimates of the errors in *k*_1_, *k*_2_, *k*_3_, and *k*_4_ stemming from the errors in the blood function. We can infer from the results that the error in the initial peak of the blood input function affects the estimation of kinetic parameter *k*_1_, *k*_3_, and *k*_4_ more than *k*_2_. The error in the tail part affects the estimation of kinetic parameter *k*_3_ most, *k*_4_ second but does not affect *k*_1_ and *k*_2_. If the overall blood input function has almost the same error rate, we find that the error in *k*_2_, *k*_3_, and *k*_4_ is negligibly small while *k*_1_ changes relatively large as apparent from equations.

The developed method can quantify the errors in the kinetic parameters for different error combinations in the blood function, without having to perform optimization for each of the error cases to be analyzed. Instead of iteratively optimizing the result of the error using the old method, we can use this analytical method to derive the ultimate error step by step. This would be computationally prohibitive especially for pixel by pixel kinetic parameter estimation, and large ranges of blood error to be analyzed. Future work includes generalization to estimation based on the sinogram instead of reconstructed TAC's and application to real PET data.

## Figures and Tables

**Table 1:** The percentage error in estimated *k*_1_, *k*_2_, *k*_3_, and *k*_4_ of liver affected by a range of erroneous blood functions in three cases.

Error rate of *C*_*p*_	Percentage error in parameters			
Case I	*k*_1_	*k*_2_	*k*_3_	*k*_4_
10%	10%	~0	~0	~0
20%	20%	~0	~0	~0

Case II	*k*_1_	*k*_2_	*k*_3_	*k*_4_
10%	10%	~0	12.5%	4%
20%	20%	~0	25%	8%

Case III	*k*_1_	*k*_2_	*k*_3_	*k*_4_
10%	~0	~0	10%	5%
20%	~0	~0	20%	10%

**Table 2:** The percentage error in estimated *k*_1_, *k*_2_, *k*_3_, and *k*_4_ of tumor affected by a range of erroneous blood functions in three cases.

Error rate of Cp	Percentage error in parameters			
Case I	*k*_1_	*k*_2_	*k*_3_	*k*_4_
10%	10%	~0	~0	~0
20%	20%	~0	~0	~0

Case II	*k*_1_	*k*_2_	*k*_3_	*k*_4_
10%	10%	~0	7.5%	25%
20%	20%	~0	15%	50%

Case III	*k*_1_	*k*_2_	*k*_3_	*k*_4_
10%	~0	~0	9%	30%
20%	~0	~0	18%	60%

## Appendix

For a solution *a*, *b*, *c*, *d* that minimizes the cost function the partial derivatives of cost function with respect to the kinetic parameters is zero

(A.1) $$\begin{array}{c} {0=\frac{\partial }{\partial a}R\left(\left[\hat{a},\hat{b},\hat{c},\hat{d}\right],{\hat{C}}_{p}\right)|}_{a=\hat{a}},\\ {0=\frac{\partial }{\partial b}R\left(\left[\hat{a},\hat{b},\hat{c},\hat{d}\right],{\hat{C}}_{p}\right)|}_{b=\hat{b}},\\ {0=\frac{\partial }{\partial c}R\left(\left[\hat{a},\hat{b},\hat{c},\hat{d}\right],{\hat{C}}_{p}\right)|}_{c=\hat{c}},\\ {0=\frac{\partial }{\partial d}R\left(\left[\hat{a},\hat{b},\hat{c},\hat{d}\right],{\hat{C}}_{p}\right)|}_{d=\hat{d}}. \end{array}$$

Let us define an implicit function which will be convenient for the application of chain rule

(A.2)
$$\begin{array}{c} \left[\hat{a},\hat{b},\hat{c},\hat{d}\right]=g\left({\hat{C}}_{p}\right)={\left[g_{1}\left({\hat{C}}_{p}\right),g_{2}\left({\hat{C}}_{p}\right),g_{3}\left({\hat{C}}_{p}\right),g_{4}\left({\hat{C}}_{p}\right)\right]}^{\prime } \end{array}$$

that maps the ${\hat{C}}_{p}$ into an estimate $\left[\hat{a},\hat{b},\hat{c},\hat{d}\right]$. We can rewrite (13) as

(A.3) $$\begin{array}{c} 0=\frac{\partial }{\partial a}R\left(g\left({\hat{C}}_{p}\right),{\hat{C}}_{p}\right),\\ 0=\frac{\partial }{\partial b}R\left(g\left({\hat{C}}_{p}\right),{\hat{C}}_{p}\right),\\ 0=\frac{\partial }{\partial c}R\left(g\left({\hat{C}}_{p}\right),{\hat{C}}_{p}\right),\\ 0=\frac{\partial }{\partial d}R\left(g\left({\hat{C}}_{p}\right),{\hat{C}}_{p}\right). \end{array}$$

By applying the chain rule to this equation, we can differentiate the above two equations with respect to ${\hat{C}}_{p}$ and obtain

(A.4) $$\begin{array}{cc} 0 & =\frac{\partial^{2}}{\partial a^{2}}R\left(g\left({\hat{C}}_{p}\right),{\hat{C}}_{p}\right)\frac{\partial }{\partial {\hat{C}}_{p}}g_{1}\left({\hat{C}}_{p}\right),\\ & \;+\frac{\partial^{2}}{\partial a\partial b}R\left(g\left({\hat{C}}_{p}\right),{\hat{C}}_{p}\right)\frac{\partial }{\partial {\hat{C}}_{p}}g_{2}\left({\hat{C}}_{p}\right),\\ & \;+\frac{\partial^{2}}{\partial a\partial c}R\left(g\left({\hat{C}}_{p}\right),{\hat{C}}_{p}\right)\frac{\partial }{\partial {\hat{C}}_{p}}g_{3}\left({\hat{C}}_{p}\right),\\ & \;+\frac{\partial^{2}}{\partial a\partial d}R\left(g\left({\hat{C}}_{p}\right),{\hat{C}}_{p}\right)\frac{\partial }{\partial {\hat{C}}_{p}}g_{4}\left({\hat{C}}_{p}\right),\\ & \;+\frac{\partial^{2}}{\partial a\partial {\hat{C}}_{p}}R\left(g\left({\hat{C}}_{p}\right),{\hat{C}}_{p}\right), \end{array}$$

and similar equations for *b*, *c*, and *d* can be obtained easily.

We can write these four equations in matrix form

(A.5) $$\begin{array}{cc} \left[\begin{array}{c} -\frac{\partial^{2}}{\partial a\partial {\hat{C}}_{p_{n}}}R\\ -\frac{\partial^{2}}{\partial b\partial {\hat{C}}_{p_{n}}}R\\ -\frac{\partial^{2}}{\partial c\partial {\hat{C}}_{p_{n}}}R\\ -\frac{\partial^{2}}{\partial d\partial {\hat{C}}_{p_{n}}}R \end{array}\right] & =\left[\begin{array}{cccc} \frac{\partial^{2}}{\partial a^{2}}R & \frac{\partial^{2}}{\partial a\partial b}R & \frac{\partial^{2}}{\partial a\partial c}R & \frac{\partial^{2}}{\partial a\partial d}R\\ \frac{\partial^{2}}{\partial b\partial a}R & \frac{\partial^{2}}{\partial b^{2}}R & \frac{\partial^{2}}{\partial b\partial c}R & \frac{\partial^{2}}{\partial b\partial d}R\\ \frac{\partial^{2}}{\partial c\partial a}R & \frac{\partial^{2}}{\partial c\partial b}R & \frac{\partial^{2}}{\partial c^{2}}R & \frac{\partial^{2}}{\partial c\partial d}R\\ \frac{\partial^{2}}{\partial d\partial a}R & \frac{\partial^{2}}{\partial d\partial b}R & \frac{\partial^{2}}{\partial d\partial c}R & \frac{\partial^{2}}{\partial d^{2}}R \end{array}\right]\\ & \;\times \left[\begin{array}{c} \frac{\partial g_{1}\left({\hat{C}}_{p}\right)}{\partial {\hat{C}}_{p_{n}}}\\ \frac{\partial g_{2}\left({\hat{C}}_{p}\right)}{\partial {\hat{C}}_{p_{n}}}\\ \frac{\partial g_{3}\left({\hat{C}}_{p}\right)}{\partial {\hat{C}}_{p_{n}}}\\ \frac{\partial g_{4}\left({\hat{C}}_{p}\right)}{\partial {\hat{C}}_{p_{n}}} \end{array}\right]. \end{array}$$

Then a simple matrix inversion provides

(A.6)
$$\begin{array}{cc} \left[\begin{array}{c} \frac{\partial g_{1}\left({\hat{C}}_{p}\right)}{\partial {\hat{C}}_{p_{n}}}\\ \frac{\partial g_{2}\left({\hat{C}}_{p}\right)}{\partial {\hat{C}}_{p_{n}}}\\ \frac{\partial g_{3}\left({\hat{C}}_{p}\right)}{\partial {\hat{C}}_{p_{n}}}\\ \frac{\partial g_{4}\left({\hat{C}}_{p}\right)}{\partial {\hat{C}}_{p_{n}}} \end{array}\right] & ={\left[\begin{array}{cccc} \frac{\partial^{2}}{\partial a^{2}}R & \frac{\partial^{2}}{\partial a\partial b}R & \frac{\partial^{2}}{\partial a\partial c}R & \frac{\partial^{2}}{\partial a\partial d}R\\ \frac{\partial^{2}}{\partial b\partial a}R & \frac{\partial^{2}}{\partial b^{2}}R & \frac{\partial^{2}}{\partial b\partial c}R & \frac{\partial^{2}}{\partial b\partial d}R\\ \frac{\partial^{2}}{\partial c\partial a}R & \frac{\partial^{2}}{\partial c\partial b}R & \frac{\partial^{2}}{\partial c^{2}}R & \frac{\partial^{2}}{\partial c\partial d}R\\ \frac{\partial^{2}}{\partial d\partial a}R & \frac{\partial^{2}}{\partial d\partial b}R & \frac{\partial^{2}}{\partial d\partial c}R & \frac{\partial^{2}}{\partial d^{2}}R \end{array}\right]}^{-1}\\ & \;\times \left[\begin{array}{c} -\frac{\partial^{2}}{\partial a\partial {\hat{C}}_{p_{n}}}R\\ -\frac{\partial^{2}}{\partial b\partial {\hat{C}}_{p_{n}}}R\\ -\frac{\partial^{2}}{\partial c\partial {\hat{C}}_{p_{n}}}R\\ -\frac{\partial^{2}}{\partial d\partial {\hat{C}}_{p_{n}}}R \end{array}\right]. \end{array}$$

The elements of the matrix and the vector on the right hand side can be calculated based on the cost function

(A.7) $$\begin{array}{cc} \frac{\partial^{2}R}{\partial a^{2}} & =2{\vec{C_{p}}}^{\prime }{\left(\frac{\partial H}{\partial a}\right)}^{\prime }\frac{\partial H}{\partial a}\vec{C_{p}},\\ \frac{\partial^{2}R}{\partial a\partial b} & =2{\vec{C_{p}}}^{\prime }{\left(\frac{\partial H}{\partial a}\right)}^{\prime }\frac{\partial H}{\partial b}\vec{C_{p}},\\ \frac{\partial^{2}R}{\partial a\partial c} & =-2{\vec{C_{p}}}^{\prime }{\left(\frac{\partial^{2}H}{\partial a\partial c}\right)}^{\prime }\vec{F}\\ & \;+2{\vec{C_{p}}}^{\prime }{\left(\frac{\partial^{2}H}{\partial a\partial c}\right)}^{\prime }H\vec{C_{p}}+2{\vec{C_{p}}}^{\prime }{\left(\frac{\partial H}{\partial a}\right)}^{\prime }\frac{\partial H}{\partial c}\vec{C_{p}},\\ \frac{\partial^{2}R}{\partial a\partial d} & =2{\vec{C_{p}}}^{\prime }{\left(\frac{\partial H}{\partial a}\right)}^{\prime }\frac{\partial H}{\partial d}\vec{C_{p}},\\ \frac{\partial^{2}R}{\partial b^{2}} & =2{\vec{C_{p}}}^{\prime }{\left(\frac{\partial H}{\partial b}\right)}^{\prime }\frac{\partial H}{\partial b}\vec{C_{p}},\\ \frac{\partial^{2}R}{\partial b\partial c} & =2{\vec{C_{p}}}^{\prime }{\left(\frac{\partial H}{\partial b}\right)}^{\prime }\frac{\partial H}{\partial c}\vec{C_{p}},\\ \frac{\partial^{2}R}{\partial b\partial d} & =-2{\vec{C_{p}}}^{\prime }{\left(\frac{\partial^{2}H}{\partial b\partial d}\right)}^{\prime }\vec{F}\\ & \;+2{\vec{C_{p}}}^{\prime }{\left(\frac{\partial^{2}H}{\partial b\partial d}\right)}^{\prime }H\vec{C_{p}}+2{\vec{C_{p}}}^{\prime }\left(\frac{\partial H}{\partial b}\right)\frac{\partial H}{\partial d}\vec{C_{p}},\\ \frac{\partial^{2}R}{\partial c^{2}} & =-2{\vec{C_{p}}}^{\prime }{\left(\frac{\partial^{2}H}{\partial c^{2}}\right)}^{\prime }\vec{F}+2{\vec{C_{p}}}^{\prime }{\left(\frac{\partial^{2}H}{\partial c^{2}}\right)}^{\prime }H\vec{C_{p}}\\ & \;+2{\vec{C_{p}}}^{\prime }{\left(\frac{\partial H}{\partial c}\right)}^{\prime }\frac{\partial H}{\partial c}\vec{C_{p}},\\ \frac{\partial^{2}R}{\partial c\partial d} & =2{\vec{C_{p}}}^{\prime }{\left(\frac{\partial H}{\partial c}\right)}^{\prime }\frac{\partial H}{\partial d}\vec{C_{p}},\\ \frac{\partial^{2}R}{\partial d^{2}} & =-2{\vec{C_{p}}}^{\prime }{\left(\frac{\partial^{2}H}{\partial d^{2}}\right)}^{\prime }\vec{F}+2{\vec{C_{p}}}^{\prime }{\left(\frac{\partial^{2}H}{\partial d^{2}}\right)}^{\prime }H\vec{C_{p}}\\ & \;+2{\vec{C_{p}}}^{\prime }{\left(\frac{\partial H}{\partial d}\right)}^{\prime }\frac{\partial H}{\partial d}\vec{C_{p}},\\ \frac{\partial^{2}R}{\partial a\partial {\hat{C}}_{p_{n}}} & =-2\left[0\cdots 01_{i=n}0\cdots 0\right]{\left(\frac{\partial H}{\partial a}\right)}^{\prime }\vec{F}\\ & \;+2\left[0\cdots 01_{i=n}0\cdots 0\right]{\left(\frac{\partial H}{\partial a}\right)}^{\prime }H\vec{C_{p}}\\ & \;+2{\vec{C_{p}}}^{\prime }{\left(\frac{\partial H}{\partial a}\right)}^{\prime }H{\left[0\cdots 01_{i=n}0\cdots 0\right]}^{\prime },\\ \frac{\partial^{2}R}{\partial b\partial {\hat{C}}_{p_{n}}} & =-2\left[0\cdots 01_{i=n}0\cdots 0\right]{\left(\frac{\partial H}{\partial b}\right)}^{\prime }\vec{F}\\ & \;+2\left[0\cdots 01_{i=n}0\cdots 0\right]{\left(\frac{\partial H}{\partial b}\right)}^{\prime }H\vec{C_{p}}\\ & \;+2{\vec{C_{p}}}^{\prime }{\left(\frac{\partial H}{\partial b}\right)}^{\prime }H{\left[0\cdots 01_{i=n}0\cdots 0\right]}^{\prime },\\ \frac{\partial^{2}R}{\partial c\partial {\hat{C}}_{p_{n}}} & =-2\left[0\cdots 01_{i=n}0\cdots 0\right]{\left(\frac{\partial H}{\partial c}\right)}^{\prime }\vec{F}\\ & \;+2\left[0\cdots 01_{i=n}0\cdots 0\right]{\left(\frac{\partial H}{\partial c}\right)}^{\prime }H\vec{C_{p}}\\ & \;+2{\vec{C_{p}}}^{\prime }{\left(\frac{\partial H}{\partial c}\right)}^{\prime }H{\left[0\cdots 01_{i=n}0\cdots 0\right]}^{\prime },\\ \frac{\partial^{2}R}{\partial d\partial {\hat{C}}_{p_{n}}} & =-2\left[0\cdots 01_{i=n}0\cdots 0\right]{\left(\frac{\partial H}{\partial d}\right)}^{\prime }\vec{F}\\ & \;+2\left[0\cdots 01_{i=n}0\cdots 0\right]{\left(\frac{\partial H}{\partial d}\right)}^{\prime }H\vec{C_{p}}\\ & \;+2{\vec{C_{p}}}^{\prime }{\left(\frac{\partial H}{\partial d}\right)}^{\prime }H{\left[0\cdots 01_{i=n}0\cdots 0\right]}^{\prime }, \end{array}$$

where “′” denotes the transpose. The terms ∂*H*/∂*a*, ∂*H*/∂*b*,∂*H*/∂*c*, ∂*H*/∂*d*, (∂^2^/∂*a*∂*c*)*H*, (∂^2^/∂*c*^2^)  *H*, (∂^2^/∂*b*∂*d*)*H*, and (∂^2^/∂*d*^2^)*H* can be calculated using the compartment model

(A.8) $$\begin{array}{cc} \frac{\partial H}{\partial a} & =\text{T}\left[e^{-ct}\right],\\ \frac{\partial H}{\partial b} & =\text{T}\left[e^{-dt}\right],\\ \frac{\partial H}{\partial c} & =\text{T}\left[-ate^{-ct}\right],\\ \frac{\partial H}{\partial d} & =\text{T}\left[-bte^{-dt}\right],\\ \frac{\partial^{2}}{\partial a\partial c}H & =\text{T}\left[-te^{-ct}\right],\\ \frac{\partial^{2}}{\partial b\partial d}H & =\text{T}\left[-te^{-dt}\right],\\ \frac{\partial^{2}}{\partial c^{2}}H & =\text{T}\left[at^{2}e^{-ct}\right],\\ \frac{\partial^{2}}{\partial d^{2}}H & =\text{T}\left[bt^{2}e^{-dt}\right], \end{array}$$

where “T” denotes an operation converting a vector to its corresponding convolution matrix.

In this paper, we modify regular Runge-Kutte methods [16] for ODE to adapt to partial differential equations. By far the most common approximation is the fourth-order Runge-Kutte approximation.

(A.9) $$\begin{array}{cc} s_{1,i} & =hu_{i}\left(C_{p_{n}},a,b,c,d\right),\\ s_{2,i} & =hu_{i}\left(C_{p_{n}}+\frac{h}{2},a+\frac{s_{1,1}}{2},b+\frac{s_{1,2}}{2},c+\frac{s_{1,3}}{2},d+\frac{s_{1,4}}{2}\right),\\ s_{3,i} & =hu_{i}\left(C_{p_{n}}+\frac{h}{2},a+\frac{s_{2,1}}{2},b+\frac{s_{2,2}}{2},c+\frac{s_{2,3}}{2},d+\frac{s_{2,4}}{2}\right),\\ s_{4,i} & =hu_{i}\left(C_{p_{n}}+h,a+s_{3,1},b+s_{3,2},c+s_{3,3},d+s_{3,4}\right),\\ y_{i}\left(C_{p_{n}}+h\right) & =y_{i}\left(C_{p_{n}}\right)+\frac{s_{1,i}}{6}+\frac{s_{2,i}}{3}+\frac{s_{3,i}}{3}+\frac{s_{4,i}}{6}, \end{array}$$

where *k*_*i*_ = *y*_*i*_(*C*_*p*_*n*__), *i* = 1,2, 3,4 for *a*, *b*, *c*, and *d*, *h* is the small step size we define according to the demand on accuracy and speed,

(A.10) $$\begin{array}{cc} u_{1} & =\frac{1}{\phi }\left[\begin{array}{cccc} -\frac{\partial^{2}}{\partial a\partial {\hat{C}}_{p_{n}}}R & \frac{\partial^{2}}{\partial a\partial b}R & \frac{\partial^{2}}{\partial a\partial c}R & \frac{\partial^{2}}{\partial a\partial d}R\\ -\frac{\partial^{2}}{\partial b\partial {\hat{C}}_{p_{n}}}R & \frac{\partial^{2}}{\partial b^{2}}R & \frac{\partial^{2}}{\partial b\partial c}R & \frac{\partial^{2}}{\partial b\partial d}R\\ -\frac{\partial^{2}}{\partial c\partial {\hat{C}}_{p_{n}}}R & \frac{\partial^{2}}{\partial c\partial b}R & \frac{\partial^{2}}{\partial c^{2}}R & \frac{\partial^{2}}{\partial c\partial d}R\\ -\frac{\partial^{2}}{\partial d\partial {\hat{C}}_{p_{n}}}R & \frac{\partial^{2}}{\partial d\partial b}R & \frac{\partial^{2}}{\partial d\partial c}R & \frac{\partial^{2}}{\partial d^{2}}R \end{array}\right],\\ u_{2} & =\frac{1}{\phi }\left[\begin{array}{cccc} \frac{\partial^{2}}{\partial a^{2}}R & -\frac{\partial^{2}}{\partial a\partial {\hat{C}}_{p_{n}}}R & \frac{\partial^{2}}{\partial a\partial c}R & \frac{\partial^{2}}{\partial a\partial d}R\\ \frac{\partial^{2}}{\partial b\partial a}R & -\frac{\partial^{2}}{\partial b\partial {\hat{C}}_{p_{n}}}R & \frac{\partial^{2}}{\partial b\partial c}R & \frac{\partial^{2}}{\partial b\partial d}R\\ \frac{\partial^{2}}{\partial c\partial a}R & -\frac{\partial^{2}}{\partial c\partial {\hat{C}}_{p_{n}}}R & \frac{\partial^{2}}{\partial c^{2}}R & \frac{\partial^{2}}{\partial c\partial d}R\\ \frac{\partial^{2}}{\partial d\partial a}R & -\frac{\partial^{2}}{\partial d\partial {\hat{C}}_{p_{n}}}R & \frac{\partial^{2}}{\partial d\partial c}R & \frac{\partial^{2}}{\partial d^{2}}R \end{array}\right],\\ u_{3} & =\frac{1}{\phi }\left[\begin{array}{cccc} \frac{\partial^{2}}{\partial a^{2}}R & \frac{\partial^{2}}{\partial a\partial b}R & -\frac{\partial^{2}}{\partial a\partial {\hat{C}}_{p_{n}}}R & \frac{\partial^{2}}{\partial a\partial d}R\\ \frac{\partial^{2}}{\partial b\partial a}R & \frac{\partial^{2}}{\partial b^{2}}R & -\frac{\partial^{2}}{\partial b\partial {\hat{C}}_{p_{n}}}R & \frac{\partial^{2}}{\partial b\partial d}R\\ \frac{\partial^{2}}{\partial c\partial a}R & \frac{\partial^{2}}{\partial c\partial b}R & -\frac{\partial^{2}}{\partial c\partial {\hat{C}}_{p_{n}}}R & \frac{\partial^{2}}{\partial c\partial d}R\\ \frac{\partial^{2}}{\partial d\partial a}R & \frac{\partial^{2}}{\partial d\partial b}R & -\frac{\partial^{2}}{\partial d\partial {\hat{C}}_{p_{n}}}R & \frac{\partial^{2}}{\partial d^{2}}R \end{array}\right],\\ u_{4} & =\frac{1}{\phi }\left[\begin{array}{cccc} \frac{\partial^{2}}{\partial a^{2}}R & \frac{\partial^{2}}{\partial a\partial b}R & \frac{\partial^{2}}{\partial a\partial c}R & -\frac{\partial^{2}}{\partial a\partial {\hat{C}}_{p_{n}}}R\\ \frac{\partial^{2}}{\partial b\partial a}R & \frac{\partial^{2}}{\partial b^{2}}R & \frac{\partial^{2}}{\partial b\partial c}R & -\frac{\partial^{2}}{\partial b\partial {\hat{C}}_{p_{n}}}R\\ \frac{\partial^{2}}{\partial c\partial a}R & \frac{\partial^{2}}{\partial c\partial b}R & \frac{\partial^{2}}{\partial c^{2}}R & -\frac{\partial^{2}}{\partial c\partial {\hat{C}}_{p_{n}}}R\\ \frac{\partial^{2}}{\partial d\partial a}R & \frac{\partial^{2}}{\partial d\partial b}R & \frac{\partial^{2}}{\partial d\partial c}R & -\frac{\partial^{2}}{\partial d\partial {\hat{C}}_{p_{n}}}R \end{array}\right], \end{array}$$

and *ϕ* is a scalar given by the determinant

(A.11) $$\phi =\left|\begin{array}{cccc} \frac{\partial^{2}}{\partial a^{2}}R & \frac{\partial^{2}}{\partial a\partial b}R & \frac{\partial^{2}}{\partial a\partial c}R & \frac{\partial^{2}}{\partial a\partial d}R\\ \frac{\partial^{2}}{\partial b\partial a}R & \frac{\partial^{2}}{\partial b^{2}}R & \frac{\partial^{2}}{\partial b\partial c}R & \frac{\partial^{2}}{\partial b\partial d}R\\ \frac{\partial^{2}}{\partial c\partial a}R & \frac{\partial^{2}}{\partial c\partial b}R & \frac{\partial^{2}}{\partial c^{2}}R & \frac{\partial^{2}}{\partial c\partial d}R\\ \frac{\partial^{2}}{\partial d\partial a}R & \frac{\partial^{2}}{\partial d\partial b}R & \frac{\partial^{2}}{\partial d\partial c}R & \frac{\partial^{2}}{\partial d^{2}}R \end{array}\right|,$$

Let us define

(A.12)
$$\begin{array}{cc} a_{i} & =y_{1}\left(C_{p_{n}}\right),\\ b_{i} & =y_{2}\left(C_{p_{n}}\right),\\ c_{i} & =y_{3}\left(C_{p_{n}}\right),\\ d_{i} & =y_{4}\left(C_{p_{n}}\right),\\ a_{i+1} & =y_{1}\left(C_{p_{n}}+h\right),\\ b_{i+1} & =y_{2}\left(C_{p_{n}}+h\right),\\ c_{i+1} & =y_{3}\left(C_{p_{n}}+h\right),\\ d_{i+1} & =y_{4}\left(C_{p_{n}}+h\right), \end{array}$$

where *h* is as defined before. In multidimension, we perform the calculations *N* times sequentially for every *C*_*p*_*n*__ for a single step

(A.13)
$$\begin{array}{cc} a_{i+1} & =a_{i}+\overset{N}{\underset{n=1}{\sum }}\frac{\partial a}{\partial {\hat{C}}_{p_{n}}}\Delta {\hat{C}}_{p_{n}},\\ b_{i+1} & =b_{i}+\overset{N}{\underset{n=1}{\sum }}\frac{\partial b}{\partial {\hat{C}}_{p_{n}}}\Delta {\hat{C}}_{p_{n}},\\ c_{i+1} & =c_{i}+\overset{N}{\underset{n=1}{\sum }}\frac{\partial c}{\partial {\hat{C}}_{p_{n}}}\Delta {\hat{C}}_{p_{n}},\\ d_{i+1} & =d_{i}+\overset{N}{\underset{n=1}{\sum }}\frac{\partial d}{\partial {\hat{C}}_{p_{n}}}\Delta {\hat{C}}_{p_{n}}. \end{array}$$
